# 1991. Q fever endocarditis is on the rise

**DOI:** 10.1093/ofid/ofac492.1616

**Published:** 2022-12-15

**Authors:** Mohamad Alhoda Mohamad Alahmad

**Affiliations:** KUMC, Overland park, Kansas

## Abstract

**Background:**

Q fever is a reportable zoonotic febrile bacterial infection caused by Coxiella burnetii. In patients who are symptomatic, acute manifestations with hepatitis, pneumonia or fever represent the majority. Elderly men and immunocompromised populations usually present with chronic disease, usually infective endocarditis (IE). Herein, we study the inpatient prevalence of Q fever.

**Methods:**

We used Nationwide Readmissions Database (NRD) and included hospitalizations of adults (≥18 years old) with a diagnosis of Q fever between January and November 2016-2019. Survey procedures were applied to accommodate for complex sampling design of NRD. Chi-square and means least-square were used for categorical and continuous variables, respectively. Jonckheere-Terpstra test was used to study the trends over the years. SAS 9.4 was used for data mining and analysis.

**Results:**

A total of 1,289 hospitalizations with a diagnosis of Q fever were included, among which 172 (13%) cases had a concurrent diagnosis of infective endocarditis. The mean age of patients was 58 years, a third were female. Our analysis demonstrated that infective endocarditis was the most common cardiac complication associated with Q fever (96%) followed by pericarditis and myopericarditis (5 and 4% respectively). There is a trend of an increase in cases of inpatient Q fever with or without endocarditis over the years (from 30 and 165 cases in 2016 to 50 and 401 cases in 2019 respectively, p-value 0.04). Although there was no significant increase in mortality or 30-day readmission between patients with or without endocarditis, patients with IE had longer inpatient stays (mean of 19 vs. 11 days, p-value < 0.001) and subsequently higher hospital charges (mean of $242,615 vs. $136,989, p-value < 0.001).

Q fever trend from 2016-2019 (%).

**Figure:**
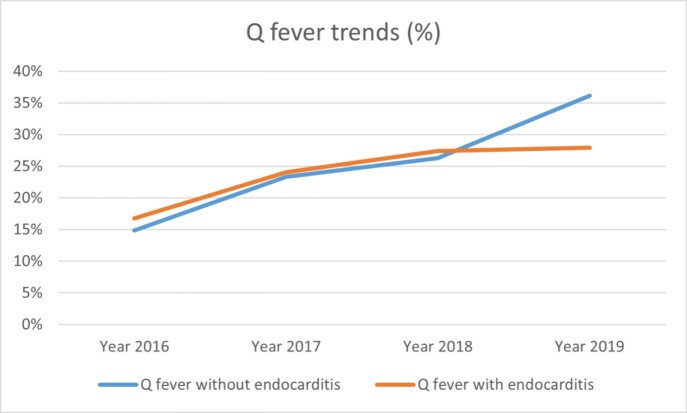

Increase in percentage of inpatient cases of Q fever with and without endocarditis across the years (cases in a year/ all cases in 2016-2019).

Q fever trend from 2016-2019.

**Figure:**
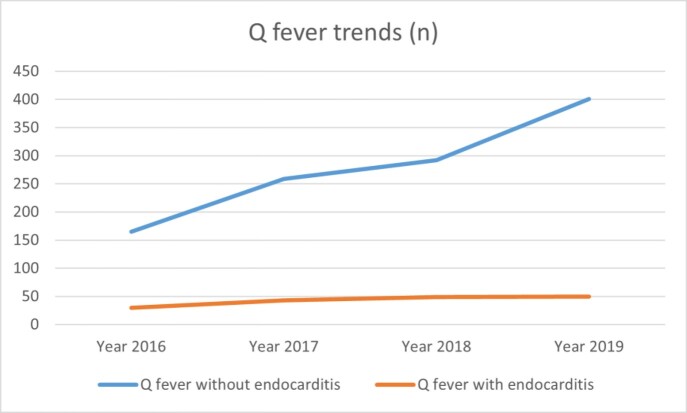

Increase in the number of inpatient cases of Q fever with and without endocarditis across the years.

**Conclusion:**

Physicians should be aware of an increasing trend of hospitalized patients with Q fever. Further studies are needed.

**Disclosures:**

**All Authors**: No reported disclosures.

